# De Novo Sequencing of a *Sparassis latifolia* Genome and Its Associated Comparative Analyses

**DOI:** 10.1155/2018/1857170

**Published:** 2018-02-25

**Authors:** Donglai Xiao, Lu Ma, Chi Yang, Zhenghe Ying, Xiaoling Jiang, Yan-quan Lin

**Affiliations:** The Institute of Edible Fungi, Fujian Academy of Agricultural Sciences, Fuzhou, Fujian 350003, China

## Abstract

Known to be rich in *β-*glucan, *Sparassis latifolia* (*S. latifolia*) is a valuable edible fungus cultivated in East Asia. A few studies have suggested that *S. latifolia* is effective on antidiabetic, antihypertension, antitumor, and antiallergen medications. However, it is still unclear genetically why the fungus has these medical effects, which has become a key bottleneck for its further applications. To provide a better understanding of this fungus, we sequenced its whole genome, which has a total size of 48.13 megabases (Mb) and contains 12,471 predicted gene models. We then performed comparative and phylogenetic analyses, which indicate that *S. latifolia* is closely related to a few species in the antrodia clade including *Fomitopsis pinicola*, *Wolfiporia cocos*, *Postia placenta*, and *Antrodia sinuosa*. Finally, we annotated the predicted genes. Interestingly, the *S. latifolia* genome encodes most enzymes involved in carbohydrate and glycoconjugate metabolism and is also enriched in genes encoding enzymes critical to secondary metabolite biosynthesis and involved in indole, terpene, and type I polyketide pathways. As a conclusion, the genome content of *S. latifolia* sheds light on its genetic basis of the reported medicinal properties and could also be used as a reference genome for comparative studies on fungi.

## 1. Introduction

*Sparassis latifolia* (*S. latifolia*), also called cauliflower mushroom, is a valuable brown-rot fungus belonging to Sparassidaceae of Polyporales. *S. latifolia* usually grows on trees like pine or larch and have a wide distribution across the Northern Temperate Zone. The mating system of *S. latifolia* is bipolar [1], and the basidiocarps are composed of numerous loosely arranged flabella that are morphologically large, broad, dissected, and slightly contorted [2].

Polysaccharides represent a major class of bioactive compounds found in mushrooms. Beta-glucan was the major bioactive component of *S. latifolia*, which composes more than 40% its dry weight [3]. Previous studies suggest that a 6-branched 1,3-beta-glucan forms the primary structure of the purified beta-glucan from this mushroom. The purified beta-glucan exhibits various biological activities, such as immune stimulation and antitumor effects [1, 3, 4]. Oral administration of *S. latifolia* also has antihypertension [5], antiallergen [6], and antidiabetic effects [7, 8]. Because of its potential in medical researches, factory cultivation of *S. latifolia* had been achieved in Japan, South Korea, and China. However, the long life-cycle and high labor intensity are still the key bottlenecks for wide cultivation.

In recent years, lots of fungal genomes were sequenced because of their importance in industry, agriculture, and medicine fields. Based on whole genomes sequencing, enzymes engaged in carbohydrate metabolism and key enzymes for secondary metabolite biosynthesis were analyzed in *Ganoderma lucidum* and *Lignosus rhinocerotis* [9–11]. In addition, Martinez et al. analyzed the lignocelluloses conversion mechanism of a brown-rot fungus *Postia placenta* using the genome, transcriptome, and secretome data [12]. They also compared it with *Phanerochaete chrysosporium*, a white-rot fungi, and identified that the function of lignin for efficient depolymerization was lost during the evolutionary shift from white-rot fungi to brown-rot ones. The genomes of a few other edible or medical mushrooms were also sequenced, for example, *Volvariella volvacea* [13], *Agaricus bisporus* [14], *Flammulina velutipes* [15], *Antrodia cinnamomea* [16], and *Wolfiporia cocos* [17].

In this study, we sequenced the whole genome of *S. latifolia*, strain “Minxiu NO.1.” To identify *S. latifolia*-specific traits, we compared its genome with other white-rot and brown-rot fungi [17]. We then performed gene function analysis and annotated genes possibly associated with lignocelluloses decomposition and mushroom formation. In addition, we studied the capacities of *S. latifolia* in producing secondary metabolites and the genes related to the biosynthesis of polysaccharides. To our best knowledge, this is the first comprehensive description and analyses on the whole genome of *S. latifolia*, a mushroom of important economical and medical values in Asia.

## 2. Results and Discussions

### 2.1. Genomic Features of *S. latifolia*

The *S. latifolia* genome was sequenced using Illumina HiSeq 2500 sequencing technologies. A total of 24,119 Mb clean genome-sequencing data (with 601X coverage) was obtained, from which 48.13 Mb draft genome was assembled (see Table 1 and Figure S1). The daft genome consists of 472 scaffolds with N50 of 640833 bp and has 51.43% G+C content. The *S. latifolia* genome is of a similar size with several other species in the order Polyporales including *Trametes versicolor* (44.79 Mb), *Wolfiporia cocos* (50.48 Mb) [17], *Phanerochaete carnosa* (46.29 Mb) [18], and *Polyporus brumalis* (45.72 Mb) (http://genome.jgi.doe.gov/Polbr1/Polbr1.info.html), but larger than the sizes of *Ganoderma* sp. (39.52 Mb) [19], *Lignosus rhinocerotis* (34.3 Mb) [11], *Fibroporia radiculosa* (28.38 Mb) [20], and *Phanerochaete chrysosporium* (35.15 Mb) [21].

We annotated the assembled genomic sequence and obtained 12,471 gene models, among which 96.19% are confirmed by RNA-seq data. Nearly 89.3% (11,147) gene models have putative biological functions, and the remaining 1324 have no apparent homology to known sequences, which are presumed to be *S. latifolia*-specific genes. Up to 11,106, 6821, 7012, and 11,026 genes have homologs with known proteins deposited in the databases NCBI nr, Pfam, SwissProt, and TrEMBL, respectively. The genome also contains 72 miRNAs (69 families), 21 rRNAs (2 families), and 115 tRNAs (47 families). Among the 115 tRNAs, eight are presumably to be possible pseudogenes, 105 are anticodon tRNAs, and the remaining 2 have undetermined anticodons.

**Figure 1:**
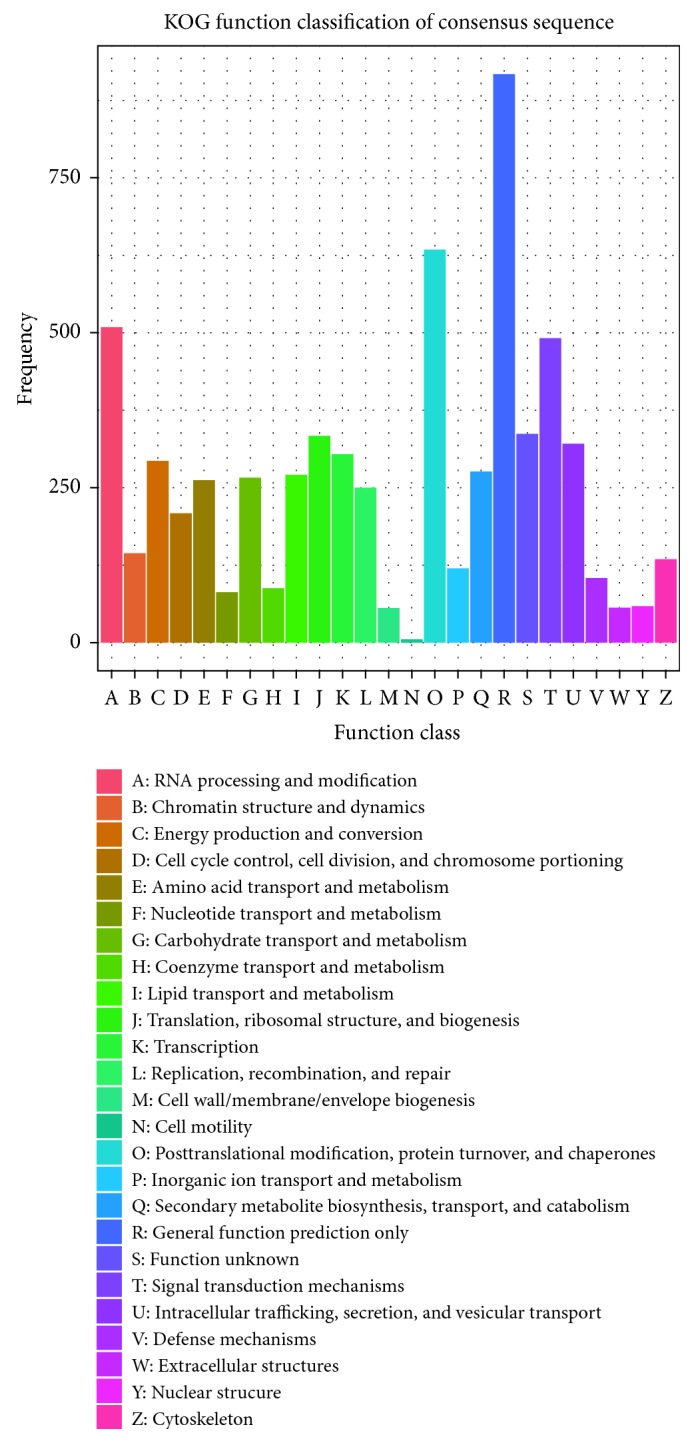
Classification of *S. latifolia* proteins by the Eukaryotic Clusters of Orthologs (KOG) database.

**Figure 2:**
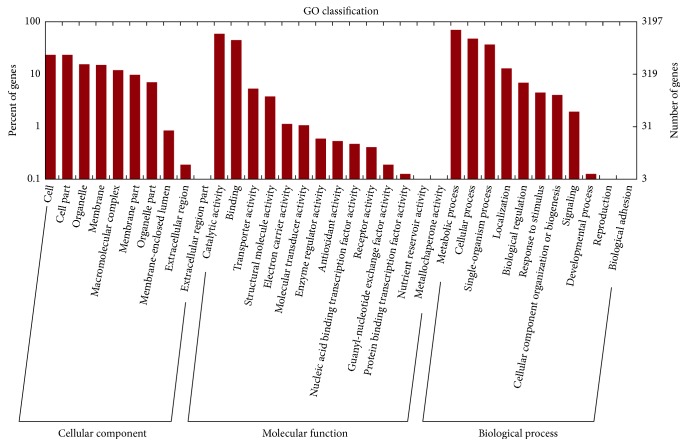
Classification of *S. latifolia* proteins by the Gene Ontology (GO) database.

**Figure 3:**
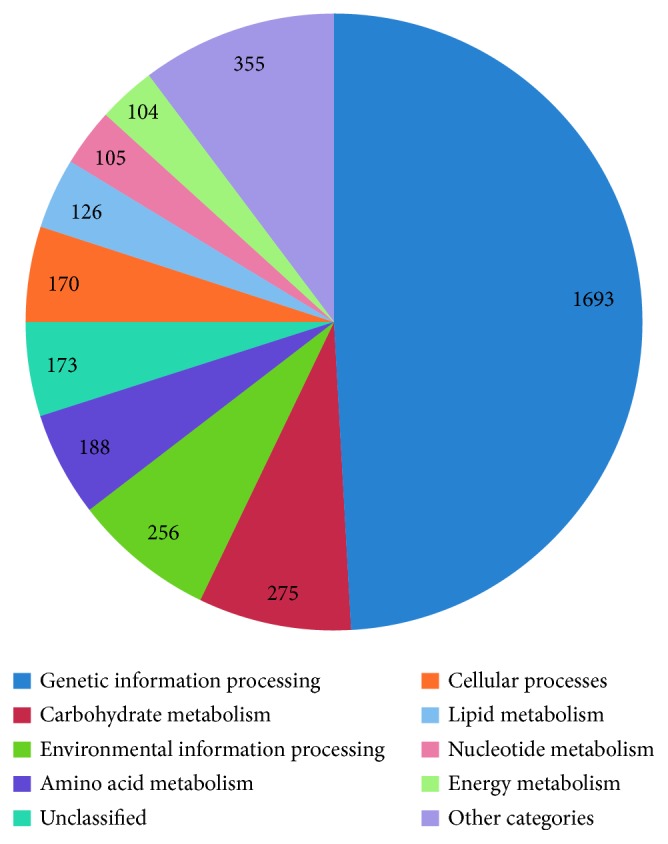
Classification of *S. latifolia* proteins by the Kyoto Encyclopedia of Genes and Genomes (KEGG) database.

In addition, we mapped the predicted genes to 3 annotation databases including Eukaryotic Clusters of Orthologs (KOG) (Figure 1), Gene Ontology (GO) (Figure 2), and the Kyoto Encyclopedia of Genes and Genomes (KEGG) (Figure 3). According to phylogenetic classification by KOGnitor, around 45.63% (5691) proteins could be assigned to KOG (Table 1). As shown in Table 1, the most enriched R category is “general functional prediction only,” which contains 917 genes. Other enriched categories include “posttranslational modification, protein turnover, chaperones” and so on. The GO analysis assigned 3197 (25.64%) proteins into different GO terms, and four categories of GO with the highest number are “catalytic activity,” “binding,” “metabolic process,” and “cellular process.” Similarly, 3445 (27.62%) putative proteins were successfully assigned to the KEGG database, and the top five pathways with the highest number include “RNA transport,” “spliceosome,” “protein processing in endoplasmic reticulum,” “purine metabolism,” and “cell cycle–yeast” (Appendix S1).

### 2.2. Protein Domain Analysis for *S. latifolia*

**Figure 4:**
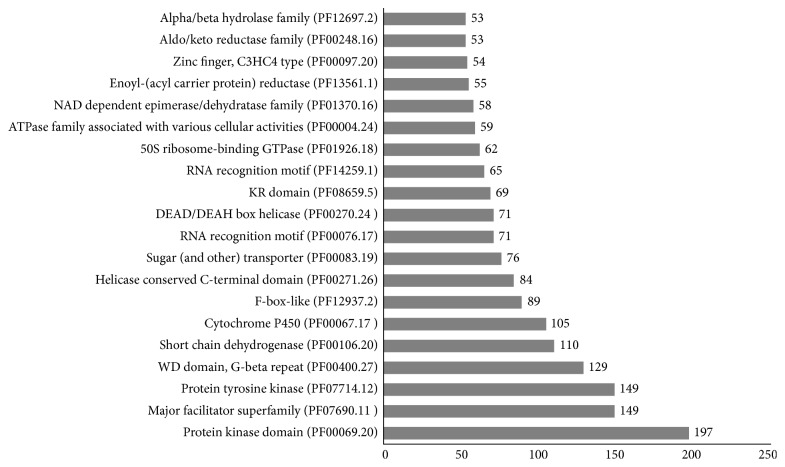
A summary for the top 20 Pfam protein domains of *S. latifolia*.

We adopted a widely used database Pfam [22] to perform protein domain analysis. In total, 6821 deduced protein sequences of *S. latifolia* were found to be associated with protein domains (Appendix S2), and the top 20 Pfam domains are plotted in Figure 4.

The top two Pfam domains are associated with protein kinase activities (197 protein kinase domains and 149 protein tyrosine kinase domains). Protein kinases have roles in every aspect of regulation and signal transduction [23]. For example, tyrosine kinase (TK) usually catalyzes the phosphorylation of Tyr residues in a protein. It is generally thought the orthologs of animal TKs are rare in fungi [24, 25]. In addition, we found 2 transporter domains including a superfamily/MSF_1 domain (PF07690.11) containing 149 proteins and a sugar (and other) transporter/sugar_tr domain (PF00083.19) containing 76 proteins. These transporters were inferred to play roles in transportation of small solutes like sugar in response to chemiosmotic ion gradients.

As we know, transcription factors help in coordinating growth, survival, or reproduction related cellular processes under certain conditions [26]. Three major transcription factor domains are PF04082.13 (39 fungal-specific transcription factor domains/Fungal_trans), PF00096.21 (39 Zinc finger, C2H2 type), and PF00172.13 (fungal Zn(2)-Cys(6) binuclear cluster domain/Zn_clus). Similar to [27], a comparison of all transcription domains suggests that the 3 TF domains are highly expanded in the selected basidiomycetes (Appendix S3).

### 2.3. Phylogenetic Analysis of *S. latifolia*

**Figure 5:**
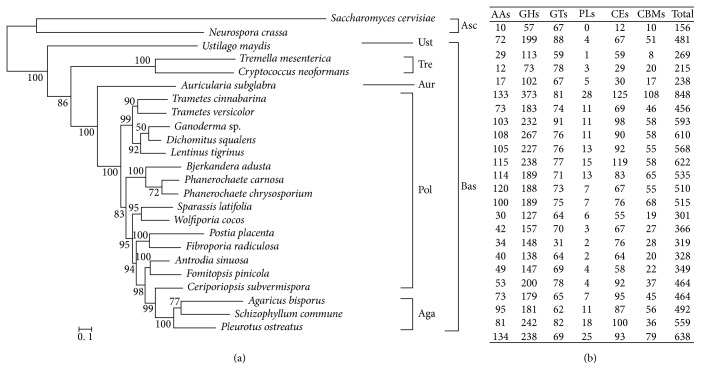
Phylogenetic tree and CAZymes analysis of *S. latifolia* and a few selected fungal species. (a) The topology of the phylogenetic tree. *Saccharomyces cerevisiae* and *Neurospora crassa* are selected as an out-group. Ust, Ustilaginales; Tre, Tremellales; Aur, Auriculariales; Pol, Polyporales; Aga, Agaricales; Asc, Ascomycota; Bas, Basidiomycota. (b) CAZymes number of *S. latifolia* with those of other fungi.

In this study, we selected 24 fungi to construct phylogenetic tree (Figure 5). Among the 24 fungi, 22 are Basidiomycota fungi, and the other two are Ascomycota fungi serving as an out-group to root the tree. Phylogenetic analysis of the single-copy orthologous proteins among the 24 fungi showed a close evolutionary relationship among *S. latifolia* to *Fomitopsis pinicola*, *Wolfiporia cocos*, *Postia placenta*, and *Antrodia sinuosa*, all of which are from the Polyporaceae family. Similar to [19], Polyporales in this study were divided into several major clades like antrodia, core polyporoid, and phlebiod clades, among which *S. latifolia* falls into the antrodia clade together with *Fomitopsis pinicola*, *Wolfiporia cocos*, *Postia placenta*, *Antrodia sinuosa*, and *Fibroporia radiculosa*, while *Ceriporiopsis subvermispora* belongs to an uncertain polyporoid clade. It is of note that additional phylogenetic information might be retrieved using phylogenetic network methods [28, 29].

### 2.4. Carbohydrate Active Enzymes (CAZymes)

As *S. latifolia* thrives on pine sawdust substrates, we mapped its genome to the CAZy database for identifying carbohydrate-active enzymes (CAZymes), carbohydrate-binding modules, and auxiliary proteins. We applied dbCAN [30] with default parameters and identified a total of 301 CAZyme-coding gene homologs (Appendix S4), which includes 127 glycoside hydrolases (GH), 64 glycosyltransferases (GT), 55 carbohydrate esterases (CE), 30 with auxiliary activities (AA), 19 carbohydrate binding module (CBM), and 6 polysaccharide lyases (PL). Interestingly, we identified lower number of CAZyme candidates than the average numbers (of CAZyme candidates) for several Basidiomycota fungi (Figure 5, Table 2).

*S. latifolia* have fewer genes encoding for the initial lignin degradation (auxiliary activities; formerly FOLymes) compared to those in the closest known brown-rot basidiomycetes such as *Fomitopsis pinicola*, *Antrodia sinuosa*, *Fibroporia radiculosa*, *Wolfiporia cocos*, and *Postia placenta* in Polyporales. Similarly, it also contains fewer genes than white-rot fungi. There are 30 AA genes in this genome including 5 AA1 (multicopper oxidases), 2 AA2 (lignin-modifying peroxidases), 11 AA3 (glucose-methanol-choline oxidoreductase including cellobiose dehydrogenase, aryl-alcohol oxidase/glucose oxidase, alcohol oxidase, pyranose oxidase), 2 AA4 (vanillyl-alcohol oxidase), 4 AA5 (copper radical oxidases), 1 AA6 (1,4-benzoquinone reductase), 3 AA7 (glucooligosaccharide oxidase), and 2 AA9 (lytic polysaccharide monooxygenase) genes. Due to their contribution in disintegration of the plant cell wall polysaccharides, the CE, GH, and PL superfamilies were also called cell wall-degrading enzymes [31], which consist mainly of cellulose, hemicellulose, and pectin [11]. However, *S. latifolia* have fewer numbers of genes coding GHs and CEs (the numbers are 127 and 55, resp.) than those of other wood-rot fungi. In addition, the number of PLs (6 genes) in *S. latifolia* genomes was the highest but was absent of CE8 (pectin methylesterase), GH89 (α-N-acetylglucosaminidase), GH78, GT41, and GT66, when compared to other five brown-rot fungi. CAZymes involved in cellulose and hemicellulose degradation were also compared (Appendix S5). Our results suggest that GH and CE genes might play weak roles in degradation of plant cell wall polysaccharides in *S. latifolia* genomes compared to other fungi.

### 2.5. Cytochrome P450 Monooxygenases

Cytochromes P450 (P450s) are heme-containing monooxygenases and widely present in species across the biological kingdoms. We retrieved the P450 genes in *S. latifolia* and 12 other Polyporales using BLAST against the P450 database (Table 3). *Phanerochaete carnosa* contains the highest number of putative P450 genes (262) followed by *Ganoderma* sp. (209), *Wolfiporia cocos* (206), and *Bjerkandera adusta* (199). However, *S. latifolia* only had a total of 105 CYPs, in which 85 CYPs can be assigned to 26 families according to Nelson's nomenclature, and the left 20 CYPs need further assignment (Appendix S6) [32]. The CYP5146 family had the largest number of genes (20 genes), followed by CYP620 (9 genes), CYP53 (7 genes), and CYP63 (6 genes) families (Table 3). CYP5146 and CYP5150 family proteins were involved in the oxidation of heterocyclic aromatic compounds, and the number of CYP5146 proteins in *S. latifolia* was highest across the selected fungi. Enrichment of CYP5146 family suggested that CYP5146 proteins might contribute to fungal adaptation to ecological niches by involving in oxidation of plant material. The gene number of the CYP620 family (involved in the secondary metabolism) was significantly higher than other selected fungi. The CYP53 family, also known as benzoate-p-hydroxylase, possibly played a key role in colonization of plants through involvement in degradation of wood [33]. *S. latifolia* also harbours six genes from the CYP63 family, which are associated with xenobiotic degradation in *Phanerochaete chrysosporium* [34]. When compared to other fungi [11], it is worth noting that *S. latifolia* has 24 genes engaged in “Metabolism of xenobiotics by cytochrome P450” and 21 genes engaged in “Drug metabolism–cytochrome P450” KEGG subpathways (Appendix S6). However, the exact roles of these CYPs are yet to be studied.

### 2.6. Secondary Metabolism

The secondary metabolism of fungi is a rich source of bioactive chemical compounds with great potential for pharmaceutical, agricultural, and nutritional applications, and secondary metabolite biosynthetic genes are often clustered [37]. There are several metabolite gene clusters in the *S. latifolia* genome, suggesting its potential in producing certain biologically active compounds (Appendix S7). There are 15 gene clusters encoding key enzymes critical to the biosynthesis of terpenes, indole, polyketides, and other secondary metabolite-related proteins. Interestingly, most of these clusters have homologous in other fungi except for clusters 1, 16, 18, and 33 (Appendix S8).

Fungal polyketides are one of the first classes of secondary metabolites and responsible for both aromatic and highly reduced polyketide metabolites [38]. The *S. latifolia* genome has 24 putative synthesis-associated genes assigned to three type I polyketide clusters. As probably the largest class of nitrogen-containing secondary metabolites, indole alkaloids are widely present in species across the biological kingdoms, many of which display potent biological activities [39]. An indole-prenyltransferase- (indole-PTase-) encoding gene was detected in cluster 16. Indole-PTase, also referred to as dimethylallyl tryptophan synthases- (DMATS-) type PTase, is one of the most common aromatic PTases in fungi. However, the indole-PTase-encoding gene in cluster 16 is not clustered with any other biosynthesis enzyme-encoding genes. In cluster 37, indole-PTase is clustered with a nonribosomal peptide synthase and PKS_ER domain. Indole precursors L-tryptophan might be directly activated by the adenylation domains of nonribosomal peptide synthetases (NRPSs).

Terpenoids is a well-recognized group of secondary metabolites for their wide usage in pharmacy. Based on anti-SMASH analysis, terpene synthase cluster was the largest cluster (located in 6 different scaffolds). The terpene synthases are known to be critical to the biosynthesis of monoterpene, sesquiterpene, and diterpene backbones [40]. A total of 4 terpene synthase genes were identified in the *S. latifolia* genome, many of which were clustered together with modifying enzymes (Appendix S7).

In addition, we identified 17 key enzymes in the mevalonate (MVA) pathway in the genome of *S. latifolia* based on KEGG. This indicates that the terpenoid backbone biosynthesis in *S. latifolia* can only proceed via the MVA pathway (Appendix S7). We list in Table 4 all of the core enzymes involved in the MVA pathway. The enzymes hydroxymethylglutaryl-CoA reductase, type III geranylgeranyl diphosphate synthase, phosphomevalonate kinase, hydroxymethylglutaryl-CoA synthase, prenylcysteine oxidase/farnesylcysteine lyase, and protein farnesyltransferase subunit beta are each coded by two copies of the genes. In contrast, the remaining 11 enzymes are encoded by a single copy of the genes. We also searched the *S. latifolia* genome for potential triterpenoid biosynthesis genes and found a gene (Gglean006755.1) that encodes lanosterol synthase (LSS; K01852; EC: 5.4.99.7). LSS was implicated in biosynthesis of the bioactive triterpenes in *Ganoderma lucidum* (ganoderic acids). The LSS in *S. latifolia* showed 73% and 81% identity to *G. lucidum* (ADD60469.1) and *Antrodia cinnamomea* (AIO10969.1), respectively. Similarly, the LSS in *S. latifolia* might be involved in biosynthesis of bioactive triterpenes. However, no bioactive triterpenes have been isolated from *S. latifolia* to date.

### 2.7. The Biosynthesis of β-Glucan

The major category of bioactive compounds found in *S. latifolia* is polysaccharide, and the most active immunomodulatory compounds are the water-soluble 1,3-*β-* and 1,6-*β-*glucans in *S. latifolia* [41]. UDP-glucose is the precursor of these glucans, whose biosynthesis involves hexokinase, phosphoglucomutase, and UTP-glucose-1-phosphate uridylyltransferase. The three enzymes are encoded by three, one, and two copies of genes, respectively, in *S. latifolia* (Table 5). In addition, *S. latifolia* encodes 2 1,3-*β-*glucan synthases and 8 *β-*glucan biosynthesis-associated proteins containing an SKN1 domain (PF03935).

**Figure 6:**
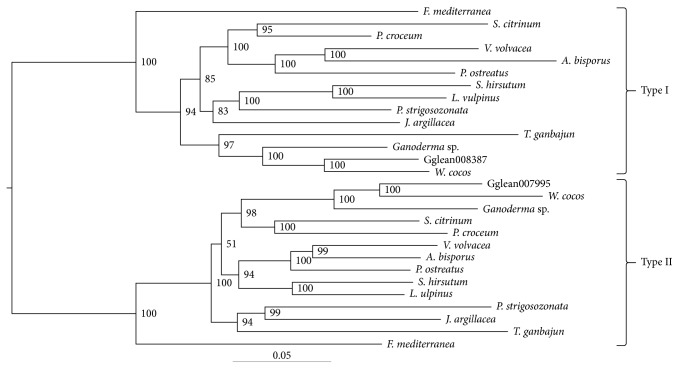
Analysis of β-glucan synthases using protein sequences in the class Agaricomycetes. Protein accession of *Fomitiporia mediterranea*, *Scleroderma citrinum*, *Piloderma croceum*, *Volvariella volvacea*, *Agaricus bisporus*, *Pleurotus ostreatus*, *Stereum hirsutum*, *Lentinellus vulpinus*, *Punctularia strigosozonata*, *Jaapia argillacea*, *Thelephora ganbajun*, *Ganoderma* sp., and *Wolfiporia cocos* in Type I (jgi|Fomme1|86578, jgi|Sclci1|1222347, jgi|Pilcr1|820169, jgi|Volvo1|118141, jgi|Agabi_varbisH97_2|226824, jgi|PleosPC9_1|114534, jgi|Stehi1|78309, jgi|Lenvul1|989499, jgi|Punst1|118018, jgi|Jaaar1|194770, jgi|Thega1|3269658, jgi|Gansp1|120993, jgi|Wolco1|84016) and Type II (jgi|Fomme1|79513, jgi|Sclci1|8244, jgi|Pilcr1|815519, jgi|Volvo1|113473, jgi|Agabi_varbisH97_2|199445, jgi|PleosPC9_1|114314, jgi|Stehi1|74453, jgi|Lenvul1|1022017, jgi|Punst1|71276, jgi|Jaaar1|125478, jgi|Thega1|3160028, jgi|Gansp1|119633, jgi|Wolco1|64852).

There are two types of 1,3-*β-*glucan synthases (i.e., Type I and II) for the mushrooms in the class Agaricomycetes [42]. Interestingly, two 1,3-*β-*glucan synthases (Gglean008387.1 and Gglean007995.1) in *S. latifolia* were also assigned to two distinct cluster (Figure 6). 1,3-*β-*glucan synthases in *S. latifolia* are integral membrane proteins. Gglean008387.1 was predicted to be consisting of 16 loops and 15 transmembrane α-helices, and Gglean007995.1 consists of 17 loops and 16 transmembrane α-helices (Appendix S9). *S. latifolia* 1,3-*β-*glucan synthases contained two catalytic domains (Fks1 and glucan synthase) and were separated by the transmembrane domain TM1. In the yeast homologue Fks1p (gi584374588), the glucan synthase domain was reported to play an important role in enzyme catalysis. Mutations in the core catalytic region of the Fks1p glucan synthase domain caused more than 30% reduction in alkali-soluble 1,3-*β-*glucan [43]. The glucan synthase domain of *S. latifolia* 1,3-*β-*glucan synthases was highly homologous to Fks1p (Appendix S10). The amino acid residues being reported to affect the catalytic activity of Fks1p were mostly conserved in both *S. latifoliaβ-*glucan synthases. *S. latifolia* produces unusually high amount of soluble 1,3-*β-*glucan, but the mechanisms are still unclear. Comparative biochemical and molecular studies with various Agaricomycetes *β-*glucan synthases may provide some explanations [42].

## 3. Materials and Methods

### 3.1. Strains and Culture Conditions

Cultivated in China, the *S. latifolia* strain “Minxiu NO.1” was provided by the Institute of Edible Fungi, Fujian Academy of Agricultural Sciences, and was grown at 25°C on PDA (20% potato, 0.2% peptone, 2% glucose, and 1.5% agar) for 25 days. To isolate genomic DNA and total RNA from mycelia, a 300 mL Erlenmeyer flask containing 50 mLPDB liquid medium (20% potato, 0.2% peptone, and 2% glucose) was inoculated with fresh plugs from the plate (five mycelial plugs/flask) and incubated at 25°C for 25 days with rotation.

### 3.2. Sequencing, Assembly, and Annotation

Using an improved cetyltrimethylammonium bromide (CTAB) method, we extracted the genomic DNA from fungal mycelium. The modified CTAB extraction buffer contained 3% (w/v) CTAB, 1.4 M NaCl, 0.1 M Tris-HCl, 5% (w/v) PVP K40, 0.02 M EDTA, and 2% (w/v) proteinase K. We then generated paired-end reads by sequencing of four cloned insert libraries of 180, 500, 3000, and 8000 bp using Hiseq 2500 system (Illumina Inc., San Diego, CA, USA) at Biomarker Technologies (Beijing, China). After that, we used the standard Illumina protocol to perform all procedures for cDNA library construction and sequencing. Raw data were processed by filtering low-quality reads by SolexaQA v2.0 (defaults to *P*=0.05, or equivalently *Q* = 13) and removing the PCR duplicates by FastUniq v1.1 with default settings. High-quality clean reads were then assembled by ALLPATHS-LG v41245 [44] with default settings. GapCloser v1.12 from SOAPdenovo2 package [45] was used to close gaps within assembled scaffolds. The protein-coding genes were predicted with a combination of Augustus v3.1, ESTs produced from transcriptome sequencing (NCBI SRA accession number: SRR3318775). Tandem repeat sequences were predicted using Tandem Repeat Finder v4.04 (parameters: Match = 2, Mismatch = 7, Delta = 7, PM = 80, PI = 10, Minscore = 50, MaxPeriod = 2000). We applied rRNA pool alignment and RNAmmer v1.2 (de novo prediction) to identify rRNA sequences, tRNAscan-SE v1.3.1 with default parameters to predict tRNA genes, and miRNAs were predicted by BLAST against mirBase 21 database (E value < 10).

To predict the functions of predicted genes, the genes were compared using BLAST against known protein and nucleotide databases (with E value < 1e-5), including the NCBI nucleotide (Nt; http://blast.ncbi.nlm.nih.gov/Blast.cgi), nonredundant set (Nr; http://blast.ncbi.nlm.nih.gov/Blast.cgi), UniProtKB (http://www.ebi.ac.uk/uniprot), Gene Ontology (GO) [46], Eukaryotic Orthologous Groups (KOGs), Clusters of Orthologous Groups (COGs) [47], Pfam [22] (http://pfam.sanger.ac.uk/), and Kyoto Encyclopedia of Genes and Genomes (KEGG; http://www.genome.jp/kegg/) protein databases [48].

### 3.3. Protein Domain Estimation

We adopted a similar procedure in Kumar et al. [49] to perform protein domain estimation of the *S. latifolia* genome. Roughly, the predicted proteins of the *S. latifolia* genome were scanned to Pfam [22] protein domain collection. Pfam domains were inferred using HMMER 3.0 [50] by removing overlapping clans. The readers were referred to [49] for detailed steps.

### 3.4. CYP and CAZy Family Classifications

*S. latifolia* protein sequences were grouped into different protein families using the National Centre for Biotechnology and Information (NCBI) Conserved Domain Database: NCBI Batch Web CD-search tool [51]. The proteins grouped under the cytochrome P450 monooxygenases superfamily were selected and aligned to fungi P450 sequences. The detected CYPs were named after the nomenclature in the P450 database, which could be found at the Cytochrome P450 homepage (http://drnelson.uthsc.edu/CytochromeP450.html) [32] or the Cytochrome P450 Engineering Database (https://cyped.biocatnet.de/) [52]. P450s that showed less than 40% identity were assigned to a new family. The dbCAN CAZyme annotation program (http://csbl.bmb.uga.edu/dbCAN/) [30] with default parameters and the Carbohydrate Active Enzymes (CAZy) database v6.0 (http://www.cazy.org) were adopted to perform the functional annotations for carbohydrate-active modules and ligninolytic enzymes, which include glycoside hydrolases (GHs), glycosyltransferases (GTs), polysaccharide lyases (PLs), carbohydrate esterases (CEs), and auxiliary activities (AAs).

### 3.5. Secondary Metabolite Gene Clusters Annotation

We first used BLAST (with E value < 1e−3) to identify putative genes encoding proteins that produce bioactive compounds. Subsequently, we analyzed the *S. latifolia* genome by antiSMASH (http://antismash.secondarymetabolites.org/) [37] to identify putative clusters, which were further examined by manually coupling with RNA-Seq data.

### 3.6. Phylogenetic Analysis

Together with *S. latifolia*, 24 fungal species mainly in the fungi divisions Basidiomycota and Ascomycota were selected for phylogenetic analysis. We obtained the genomic data of 5 species (i.e., *Ganoderma* sp., *Lentinus tigrinus*, *Bjerkandera adusta*, *Phanerochaete chrysosporium*, and *Antrodia sinuosa*) from the Joint Genome Institute (JGI) and those for 18 other species (i.e., *Ceriporiopsis subvermispora*, *Fibroporia radiculosa*, *Fomitopsis pinicola*, *Wolfiporia cocos*, *Postia placenta*, *Phanerochaete carnosa*, *Trametes versicolor*, *Dichomitus squalens*, *Trametes cinnabarina*, *Cryptococcus neoformans*, *Ustilago maydis*, *Neurospora crassa*, *Saccharomyces cerevisiae*, *Schizophyllum commune*, *Pleurotus ostreatus*, *Agaricus bisporus*, *Auricularia delicate*, and *Tremella mesenterica*) from NCBI. In addition, we also used our customized Perl program to select the longest transcript of each gene as candidate data. The orthologues were clustered by comparison of protein data sets among 24 species and the blastall program with parameters “-p blastp - -m 8 -e 1e-7” and the OrthoMCL 5 [53] program with default parameters. Phylogenetic tree were constructed by RAxML-7.2.8-ALPHA [54] with parameters “-m GTRGAMMA -# 20” and bootstrap test 1000 times.

Protein sequences of *β-*glucan synthases from the different species were aligned using MUSCLE 3.6 [55, 56]. The multiple sequence alignments were concatenated upon removing poorly aligned regions by the GBlocks server [57]. We then used a software PROTTEST 3.4 [58] to select the best model to fit protein evolution of the concatenated alignment. Phylogenetic analysis was conducted with Bayesian inference (BI) implemented in MrBayes v3.2.5 [59] under the LG *+* G *+* I model.

## Figures and Tables

**Table 1:** Summary statistics of the *S. latifolia* genome.

Sequence and assembly	Statistics
Scaffold number	472
Scaffold length (Mb)	48.13
Scaffold N50 (Kb)	640.83
GC content (%)	51.43
Length of classified repeats (%)	5.19 Mb (10.79%)
Number of predicted gene models	12,471
Average transcript length (bp)	1216
Average number of exons per gene	4.9
Average exon size (bp)	246
Average intron size (bp)	84
Number of tRNA genes	115
Number of rRNA genes	21
Number of miRNA genes	72

Gene prediction	Number

NR annotation	11,106
KEGG annotation	3445
KOG annotation	5691
COG annotation	3919
GO annotation	3197
Pfam annotation	6821
Swissport annotation	7012
TrEMBL annotation	11,026

**Table 2:** Comparison of the number of AAs family of *S. latifolia* with those of other fungi.

		AA1	AA2	AA3	AA4	AA5	AA6	AA7	AA8	AA9	AA10	AA11	AA12	AA13
Brown rot	*S. latifolia*	5	2	11	2	4	1	3	0	2	0	0	0	0
	*P. placenta*	5	1	18	0	1	0	5	0	4	0	0	0	0
	*F. pinicola*	6	2	20	1	4	1	14	0	4	0	1	0	0
	*F. radiculosa*	4	2	22	0	3	1	5	0	2	0	1	0	0
	*A. sinuosa*	7	2	22	3	4	1	7	0	2	0	1	0	0
	*W. cocos*	4	2	15	1	4	1	12	0	2	0	1	0	0
White rot	*T. versicolor*	8	27	23	3	9	1	11	2	18	0	1	0	0
	*L. tigrinus*	10	23	29	2	10	2	20	2	16	0	1	0	0
	*P. chrysosporium*	2	17	37	1	7	4	13	2	16	0	0	1	0
	*T. cinnabarina*	7	11	23	1	7	1	5	1	17	0	0	0	0
	*A. subglabra*	7	22	45	1	8	4	15	1	20	0	6	4	0
	*P. carnosa*	8	12	49	1	6	4	24	2	11	0	0	3	0
	*Ganoderma* sp.	18	11	34	1	9	2	14	1	16	0	2	0	0
	*P. ostreatus*	12	9	42	1	15	2	23	1	28	0	0	1	0
	*D. squalens*	12	14	36	3	9	1	11	2	15	0	2	0	0
	*S. commune*	3	2	23	2	2	4	12	1	22	0	10	0	0
	*B. adusta*	2	21	38	1	7	4	10	2	27	0	1	1	0
	*C. subvermispora*	9	17	22	1	3	0	10	2	9	0	0	0	0
	*A. bisporus*	13	5	36	1	9	4	14	1	11	0	0	1	0
	*N. crassa*	10	6	11	3	2	1	18	0	13	0	4	3	1
	*T. mesenterica*	4	0	2	0	2	1	2	0	0	0	1	0	0
	*C. neoformans*	4	2	2	1	3	2	1	0	1	0	1	0	0
	*U. maydis*	3	3	10	1	4	1	6	0	0	1	0	0	0
	*S. cerevisiae*	2	1	0	3	0	3	1	0	0	0	0	0	0

**Table 3:** CYP450 genes identified in fungal species.

	Fungal species	P450 count	Reference
Brown rot	*Sparassis latifolia*	105	This study
	*Fibroporia radiculosa*	176	This study
	*Fomitopsis pinicola*	190	[17]
	*Laetiporus sulphureus*	167	This study
	*Postia placenta*	190	[35]
	*Wolfiporia cocos*	206	[17]
White rot	*Trametes versicolor*	190	[17]
	*Dichomitus squalens*	187	[17]
	*Lentinus tigrinus*	194	This study
	*Phanerochaete carnosa*	262	[36]
	*Bjerkandera adusta*	199	[35]
	*Ganoderma* sp.	209	[35]
	*Phanerochaete chrysosporium*	161	[35]

**Table 4:** A list of putative genes involved in terpenoid backbone biosynthesis.

Gene name and definition	EC no.	KO term	Gene ID
Hydroxymethylglutaryl-CoA reductase	1.1.1.34	K00021	Gglean000823.1, Gglean000824.1
Protein-S-isoprenylcysteine O-methyltransferase	2.1.1.100	K00587	Gglean010277.1
Acetyl-CoA C-acetyltransferase	2.3.1.9	K00626	Gglean006582.1
Farnesyl diphosphate synthase	2.5.1.1 2.5.1.10	K00787	Gglean006352.1
Geranylgeranyl diphosphate synthase, type III	2.5.1.1 2.5.1.10 2.5.1.29	K00804	Gglean002785.1, Gglean011737.1
Phosphomevalonate kinase	2.7.4.2	K00938	Gglean000456.1, Gglean000457.1
Diphosphomevalonate decarboxylase	4.1.1.33	K01597	Gglean011667.1
Hydroxymethylglutaryl-CoA synthase	2.3.3.10	K01641	Gglean007166.1, Gglean007167.1
Isopentenyl-diphosphate delta-isomerase	5.3.3.2	K01823	Gglean001358.1
Hexaprenyl-diphosphate synthase	2.5.1.82 2.5.1.83	K05355	Gglean004204.1
Prenylcysteine oxidase/farnesylcysteine lyase	1.8.3.5 1.8.3.6	K05906	Gglean009234.1, Gglean010460.1
Protein farnesyltransferase subunit beta	2.5.1.58	K05954	Gglean006402.1, Gglean006403.1
Protein farnesyltransferase/geranylgeranyltransferase type-1 subunit alpha	2.5.1.58 2.5.1.59	K05955	Gglean005496.1
STE24 endopeptidase	3.4.24.84	K06013	Gglean006227.1
Prenyl protein peptidase	3.4.22.-	K08658	Gglean002780.1
Ditrans,polycis-polyprenyl diphosphate synthase	2.5.1.87	K11778	Gglean010851.1
Dehydrodolichyl diphosphate synthase complex subunit NUS1	2.5.1.87	K19177	Gglean001412.1

**Table 5:** Genes involved in polysaccharide biosynthesis.

Gene ID	KO/Pfam ID	Gene description
Gglean003196.1	K00844	Hexokinase [EC:2.7.1.1]
Gglean003197.1	K00844	Hexokinase [EC:2.7.1.1]
Gglean005897.1	K00844	Hexokinase [EC:2.7.1.1]
Gglean005877.1	K01835	Phosphoglucomutase [EC:5.4.2.2]
Gglean000263.1	K00963	UTP–glucose-1-phosphate uridylyltransferase [EC:2.7.7.9]
Gglean000264.1	K00963	UTP–glucose-1-phosphate uridylyltransferase [EC:2.7.7.9]
Gglean008387.1	K00706	1,3-Beta-glucan synthase [EC:2.4.1.34]
Gglean007995.1	K00706	1,3-Beta-glucan synthase [EC:2.4.1.34]
Gglean003497.1	PF03935	Beta-glucan synthesis-associated protein (SKN1)
Gglean005254.1	PF03935	Beta-glucan synthesis-associated protein (SKN1)
Gglean005257.1	PF03935	Beta-glucan synthesis-associated protein (SKN1)
Gglean008218.1	PF03935	Beta-glucan synthesis-associated protein (SKN1)
Gglean008219.1	PF03935	Beta-glucan synthesis-associated protein (SKN1)
Gglean009159.1	PF03935	Beta-glucan synthesis-associated protein (SKN1)
Gglean009770.1	PF03935	Beta-glucan synthesis-associated protein (SKN1)
Gglean001709.1	PF03935	Beta-glucan synthesis-associated protein (SKN1)

## Data Availability

This Whole Genome Shotgun project has been deposited at DDBJ/ENA/GenBank under the accession LWKX00000000. The version described in this paper is version LWKX01000000. Additionally, more data can be downloaded from our institute website: http://www.fj-mushroom.cn/Sparassis%20latifolia%20genome/1.rar.

## Supplementary Materials

Supplementary 1 Figure S1: Venn diagram of gene prediction number.

Supplementary 2 Appendix S1: KEGG pathway classification of *S. latifolia*.

Supplementary 3 Appendix S2: Pfam analysis on the protein domains of *S. latifolia*.

Supplementary 4 Appendix S3: comparing protein-coding domains of *S. latifolia* with other species.

Supplementary 5 Appendix S4: results of dbCAN analysis.

Supplementary 6 Appendix S5: comparing CAZymes involved in cellulose and hemicellulose degradation.

Supplementary 7 Appendix S6: analysis against P450 database.

Supplementary 8 Appendix S7: secondary metabolite gene clusters of *S. latifolia*.

Supplementary 9 Appendix S8: identifying homologous for gene clusters.

Supplementary 10 Appendix S9: two 1,3-*β-*glucan synthases Gglean008387.1 and Gglean007995.1.

Supplementary 11 Appendix S10: the glucan synthase domain of *S. latifolia* 1,3-*β-*glucan synthases were highly homologous to Fks1p.
