# Supplementary figures and images for: De Novo Sequencing of a Sparassis latifolia Genome and Its Associated Comparative Analyses
Source: Can J Infect Dis Med Microbiol. 2018 Feb 25;2018:1857170. doi: 10.1155/2018/1857170 (PMC5845502; doi:10.1155/2018/1857170)

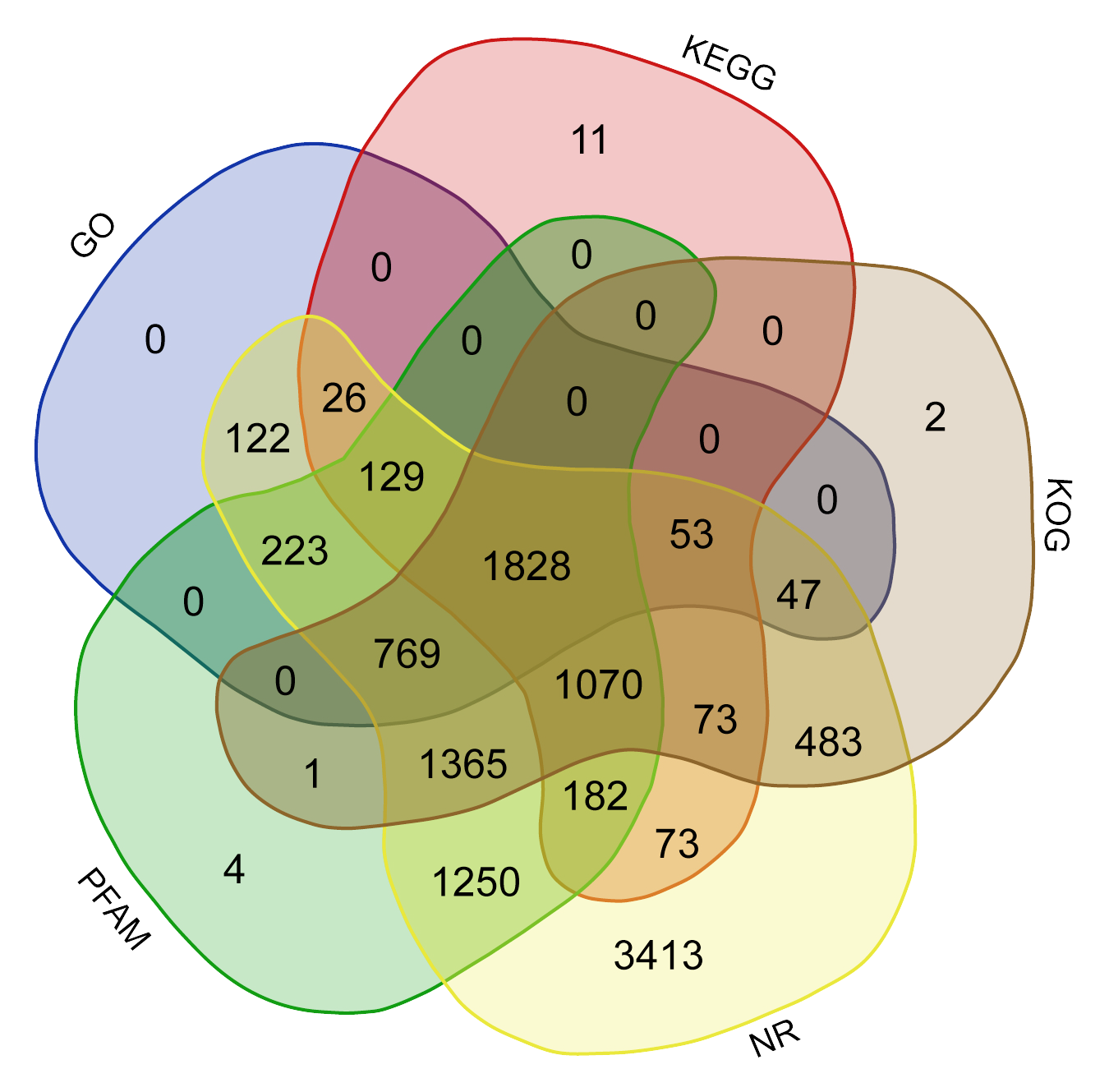

Supplement: Supplementary 1 — Figure S1: Venn diagram of gene prediction number. [file 1857170.f1.tif]
