# Supplementary material for: De Novo Sequencing of a Sparassis latifolia Genome and Its Associated Comparative Analyses
Source: Can J Infect Dis Med Microbiol. 2018 Feb 25;2018:1857170. doi: 10.1155/2018/1857170 (PMC5845502; doi:10.1155/2018/1857170)

Genes are color coded by predicted function. Putative biosynthetic genes are colored red, transport-related genes are colored blue, and regulation-related genes are colored green.

**scaffold_0 - Gene Cluster 1. Type = terpene. Location: 882819 - 903687 nt**


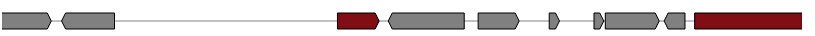


**scaffold_1 - Gene Cluster 6. Type = other. Location: 1319909 - 1489114 nt**


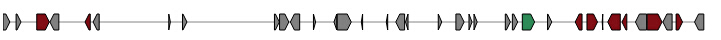


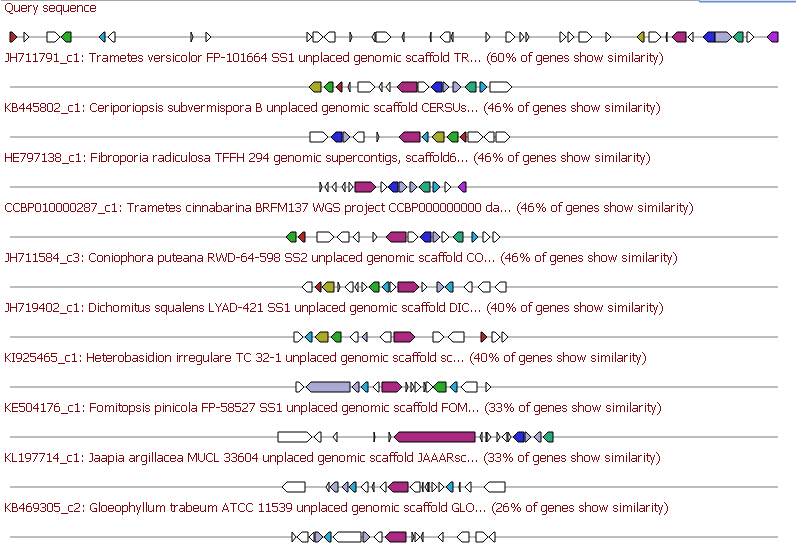


**scaffold_9 - Gene Cluster 16. Type = indole. Location: 505667 - 527021 nt**


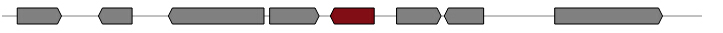


**scaffold_9 - Gene Cluster 17. Type = terpene. Location: 771304 - 906934 nt.**


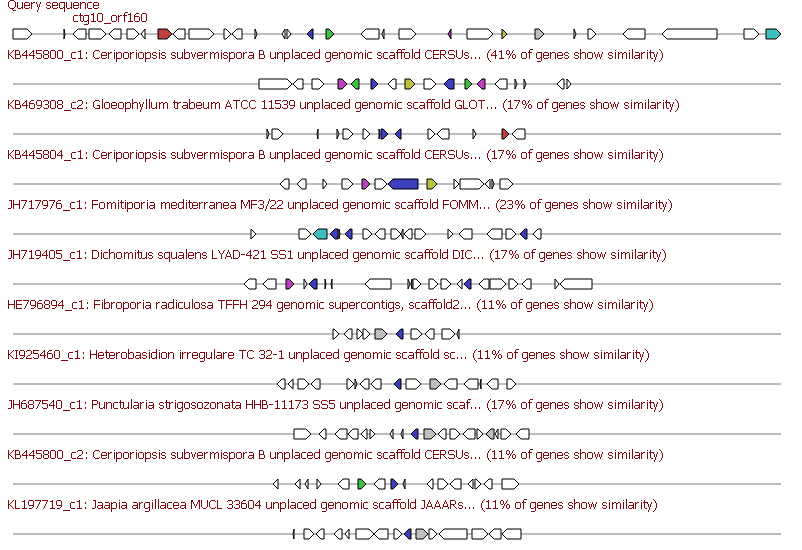


**scaffold_10 - Gene Cluster 18. Type = other. Location: 315516 - 360374 nt**


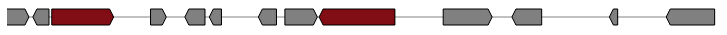


**scaffold_12 - Gene Cluster 20. Type = t1pks. Location: 286629 - 352776 nt.**


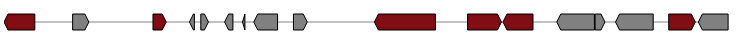


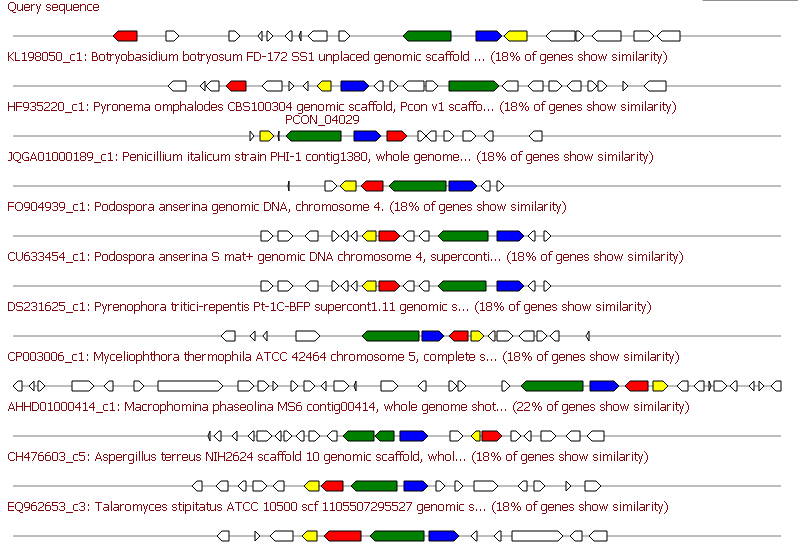


**scaffold_16 - Gene Cluster 23. Type = terpene. Location: 473054 - 580510 nt**


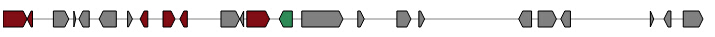


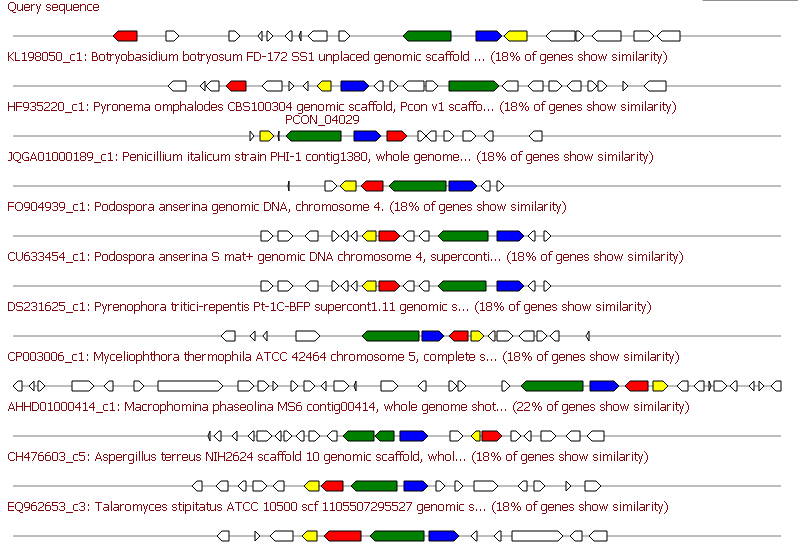


**scaffold_28 - Gene Cluster 33. Type = other. Location: 54271 - 99393 nt.**


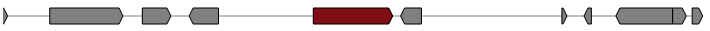


**scaffold_31 - Gene Cluster 36. Type = terpene. Location: 199275 - 219507 nt.**


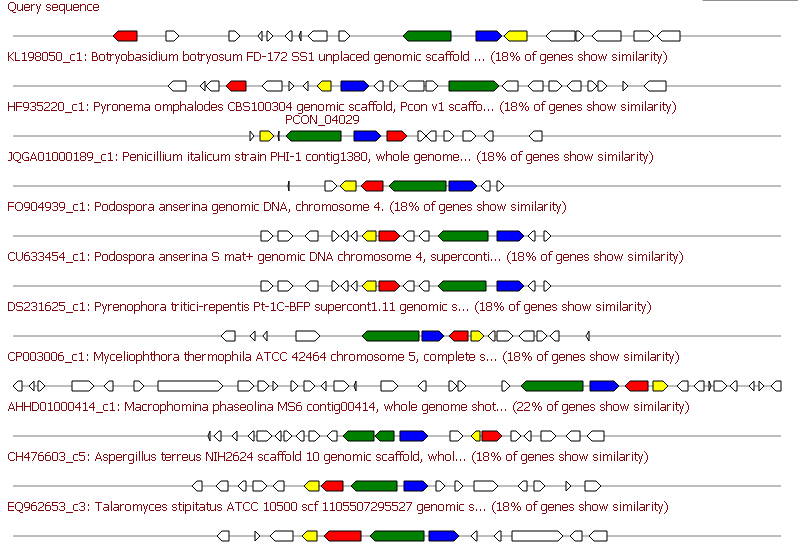


**scaffold_32 - Gene Cluster 37. Type = nrps-indole. Location: 54640 - 220594 nt.**


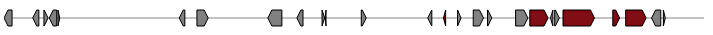


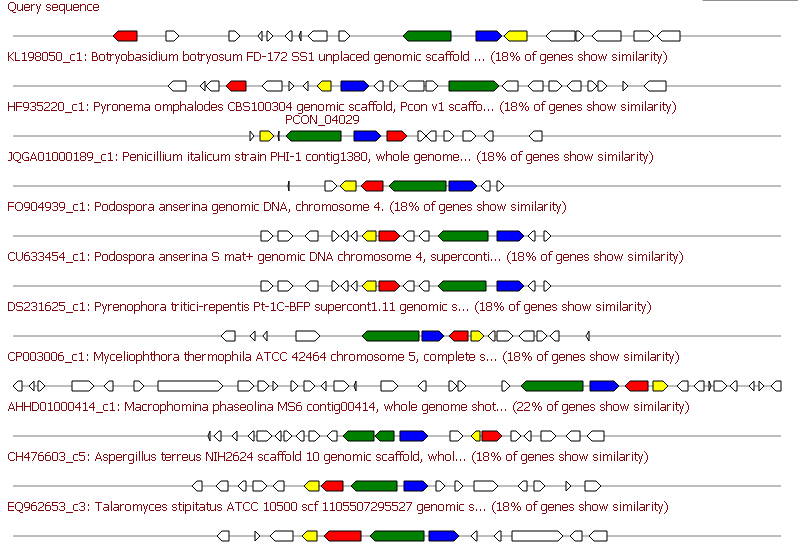


**scaffold_32 - Gene Cluster 38. Type = t1pks. Location: 248673 - 394761 nt.**


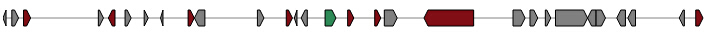


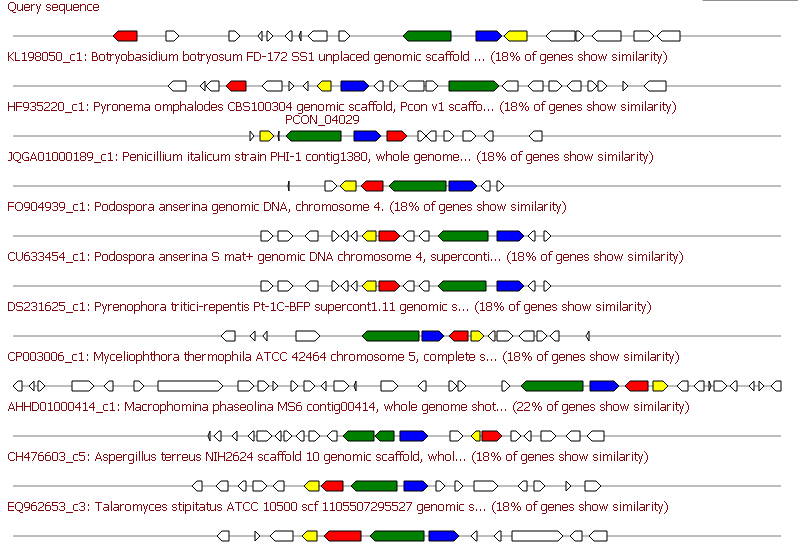


**scaffold_34 - Gene Cluster 39. Type = t1pks. Location: 277 - 395232 nt.**


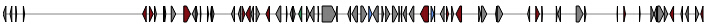


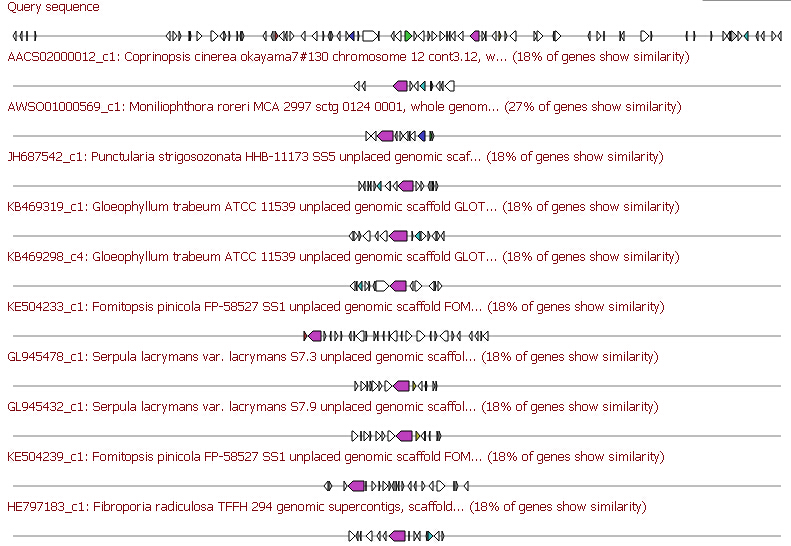


**scaffold_37 - Gene Cluster 40. Type = other. Location: 330624 - 374291 nt**


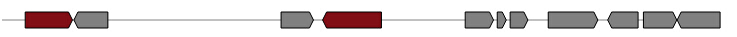


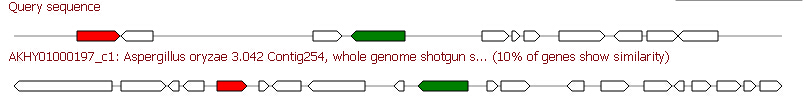


**scaffold_44 - Gene Cluster 41. Type = terpene. Location: 88755 - 109892 nt.**


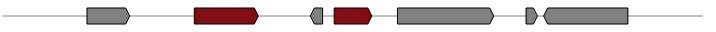


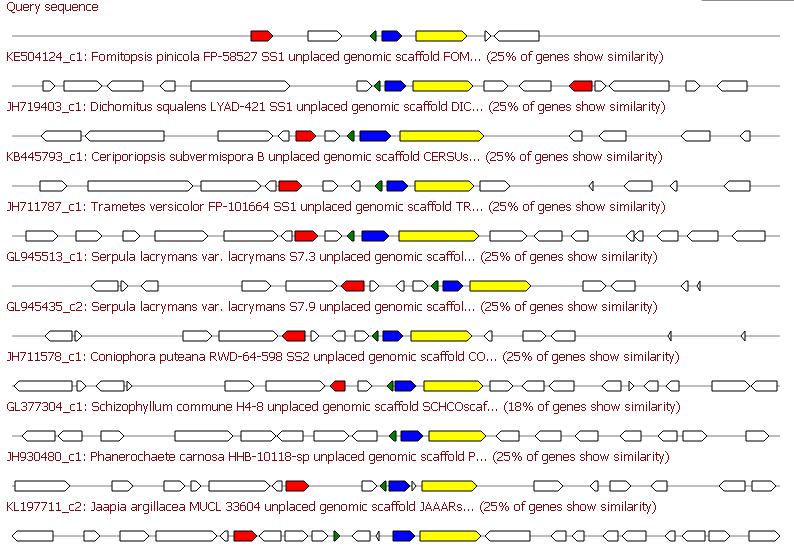


**scaffold_55 - Gene Cluster 48. Type = terpene. Location: 113439 - 134767 nt.**


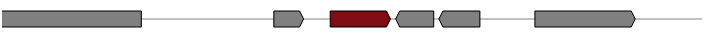


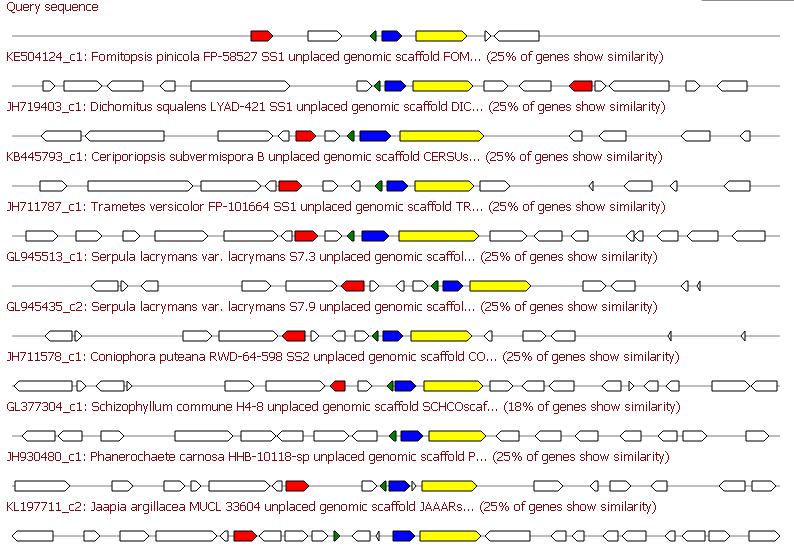

Supplement: Supplementary 9 — Appendix S8: identifying homologous for gene clusters. [file 1857170.f9.docx]
